# Supplementary material for: Deep learning-driven fragment ion series classification enables highly precise and sensitive de novo peptide sequencing
Source: Nat Commun. 2024 Jan 2;15:151. doi: 10.1038/s41467-023-44323-7 (PMC10762064; doi:10.1038/s41467-023-44323-7)
Supplement: Supplementary file 2 — Reporting Summary [file 41467_2023_44323_MOESM2_ESM.pdf]

nature portfolio

Corresponding author(s): Julien Gagneur
Mathias Wilhelm

Last updated by author(s): Nov 23, 2023

Reporting Summary

Nature Portfolio wishes to improve the reproducibility of the work that we publish. This form provides structure for consistency and transparency in reporting. For further information on Nature Portfolio policies, see our Editorial Policies and the Editorial Policy Checklist.

Statistics

For all statistical analyses, confirm that the following items are present in the figure legend, table legend, main text, or Methods section.

n/a	Confirmed	
☐	☒	The exact sample size (n) for each experimental group/condition, given as a discrete number and unit of measurement
☐	☒	A statement on whether measurements were taken from distinct samples or whether the same sample was measured repeatedly
☐	☒	The statistical test(s) used AND whether they are one- or two-sided<br>*Only common tests should be described solely by name; describe more complex techniques in the Methods section.*
☐	☒	A description of all covariates tested
☒	☐	A description of any assumptions or corrections, such as tests of normality and adjustment for multiple comparisons
☐	☒	A full description of the statistical parameters including central tendency (e.g. means) or other basic estimates (e.g. regression coefficient) AND variation (e.g. standard deviation) or associated estimates of uncertainty (e.g. confidence intervals)
☐	☒	For null hypothesis testing, the test statistic (e.g. F, t, r) with confidence intervals, effect sizes, degrees of freedom and P value noted<br>*Give P values as exact values whenever suitable.*
☒	☐	For Bayesian analysis, information on the choice of priors and Markov chain Monte Carlo settings
☒	☐	For hierarchical and complex designs, identification of the appropriate level for tests and full reporting of outcomes
☐	☒	Estimates of effect sizes (e.g. Cohen's d, Pearson's r), indicating how they were calculated

Our web collection on statistics for biologists contains articles on many of the points above.

Software and code

Policy information about availability of computer code

Data collection	MaxQuant (version 1.5.3.30), Casanovo (v2.0.0, v3.2.0), Novor (v1.05), DeepNovo (v0.0.1), PointNovo (v0.0.1)
Data analysis	Spectralis (https://github.com/gagneurlab/spectralis), blastp (version 2.12.0+), STAR (v2.7.10a), DROP (v1.2.2), pyteomics (v4.6), PyTorch (v1.8.1), Prosit, scikit-learn (v0.24.2), editdistance (https://github.com/roy-ht/editdistance, v0.5.3), XGBoost (v1.6.2), optuna (v2.8.0)

For manuscripts utilizing custom algorithms or software that are central to the research but not yet described in published literature, software must be made available to editors and reviewers. We strongly encourage code deposition in a community repository (e.g. GitHub). See the Nature Portfolio guidelines for submitting code & software for further information.

Data

Policy information about availability of data

All manuscripts must include a data availability statement. This statement should provide the following information, where applicable:

- Accession codes, unique identifiers, or web links for publicly available datasets
- A description of any restrictions on data availability
- For clinical datasets or third party data, please ensure that the statement adheres to our policy

The mass spectrometric raw data from the human dataset by Wang et al. including the MaxQuant Spectronaut search data is available via the PRIDE database with the dataset identifier PXD010154 [https://www.ebi.ac.uk/pride/archive/projects/PXD010154]. RNA-Seq data is available in the following database: ArrayExpress E-MTAB-2836 [http://www.ebi.ac.uk/arrayexpress/experiments/E-MTAB-2836/]. The human proteome database (genome build GRCh38, release 83) was downloaded from Ensembl [https://ftp.ensembl.org/pub/release-83/fasta/homo_sapiens/pep/].

The raw mass spectrometric data for the nine-species dataset by Tran et al. is available via the PRIDE database with identifiers: PXD005025 [https://www.ebi.ac.uk/pride/archive/projects/PXD005025], PXD004948 [https://www.ebi.ac.uk/pride/archive/projects/PXD004948], PXD004325 [https://www.ebi.ac.uk/pride/archive/projects/PXD004325], PXD004565 [https://www.ebi.ac.uk/pride/archive/projects/PXD004565], PXD004536 [https://www.ebi.ac.uk/pride/archive/projects/PXD004536], PXD004947 [https://www.ebi.ac.uk/pride/archive/projects/PXD004947], PXD003868 [https://www.ebi.ac.uk/pride/archive/projects/PXD003868], PXD004467 [https://www.ebi.ac.uk/pride/archive/projects/PXD004467], and PXD004424 [https://www.ebi.ac.uk/pride/archive/projects/PXD004424]. The correct peptide identifications, as well as predictions by DeepNovo, can be downloaded from the MassIVE repository with identifier MSV000081382 [https://massive.ucsd.edu/ProteoSAFe/dataset.jsp?task=b7789710a31f488c9a74eb3e3b6f61eb].

Model weights for running Casanovo were downloaded from Zenodo with DOI zenodo.6791263 [https://zenodo.org/badge/DOI/10.5281/zenodo.6791263]60.

The trained bin reclassification model and random forest, as well as Novor, Casanovo, DeepNovo, PointNovo and Spectralis predicted peptides with respective scores are deposited on Zenodo with DOI zenodo.8393846 [https://zenodo.org/record/8393846].

The data to reproduce the main figures in this study have been deposited in the Figshare repository with DOI figshare.23536794 [https://doi.org/10.6084/m9.figshare.23536794].

Source data are provided with this paper.

Research involving human participants, their data, or biological material

Policy information about studies with human participants or human data. See also policy information about sex, gender (identity/presentation), and sexual orientation and race, ethnicity and racism.

Reporting on sex and gender	Sex and gender information were not considered in the study design.
Reporting on race, ethnicity, or other socially relevant groupings	Race, etnicity or other socially relevant groupings were not considered in the study design.
Population characteristics	Population characteristics were not considered in the study design.
Recruitment	No raw mass spectrometry data nor RNA-seq data was generated but only downloaded from the sources mentioned in the data availability statement.
Ethics oversight	Technical University Munich

Note that full information on the approval of the study protocol must also be provided in the manuscript.

Field-specific reporting

Please select the one below that is the best fit for your research. If you are not sure, read the appropriate sections before making your selection.

[x] Life sciences [ ] Behavioural & social sciences [ ] Ecological, evolutionary & environmental sciences

For a reference copy of the document with all sections, see nature.com/documents/nr-reporting-summary-flat.pdf

Life sciences study design

All studies must disclose on these points even when the disclosure is negative.

Sample size	We used all data from Wang et al. 7,902,759 experimental spectra across 30 healthy human tissues downloaded from PRIDE with identifier PXD010154 [https://www.ebi.ac.uk/pride/archive/projects/PXD010154] for training our models.
Data exclusions	No data exclusion was performed.
Replication	We replicated our findings in silico: the replicates consists of the measurements on 30 different human tissues . We observed a successful replication of the results across the 30 tissues. We also replicated our findings on an independent dataset across 9 species.
Randomization	We randomly split the dataset into train, validation and test set.
Blinding	Blinding was not relevant to this study because there is no expected observer bias.

Reporting for specific materials, systems and methods

We require information from authors about some types of materials, experimental systems and methods used in many studies. Here, indicate whether each material, system or method listed is relevant to your study. If you are not sure if a list item applies to your research, read the appropriate section before selecting a response.

Materials & experimental systems

n/a	Involved in the study
☒	☐ Antibodies
☒	☐ Eukaryotic cell lines
☒	☐ Palaeontology and archaeology
☒	☐ Animals and other organisms
☒	☐ Clinical data
☒	☐ Dual use research of concern
☒	☐ Plants

Methods

n/a	Involved in the study
☒	☐ ChIP-seq
☒	☐ Flow cytometry
☒	☐ MRI-based neuroimaging

Plants

Seed stocks: Not applicable.

Novel plant genotypes: Not applicable.

Authentication: Not applicable.
