# Supplementary material for: Deep learning-driven fragment ion series classification enables highly precise and sensitive de novo peptide sequencing
Source: Nat Commun. 2024 Jan 2;15:151. doi: 10.1038/s41467-023-44323-7 (PMC10762064; doi:10.1038/s41467-023-44323-7)
Supplement: Supplementary file 3 — Supplementary Information [file 41467_2023_44323_MOESM3_ESM.pdf]

Deep learning-driven fragment ion series classification enables highly precise and sensitive de novo peptide sequencing

- Supplementary Information

Daniela Klaproth-Andrade[1,2], Johannes Hingerl[1], Yanik Bruns[1], Nicholas H. Smith[1], Jakob Träuble[1], Mathias Wilhelm[3,*], Julien Gagneur[1,2,4,5,*]

[1] Computational Molecular Medicine, School of Computation, Information and Technology, Technical University of Munich, Munich, Germany
[2] Munich Data Science Institute, Technical University of Munich, Garching, Germany
[3] Computational Mass Spectrometry, School of Life Sciences, Technical University of Munich, Munich, Germany
[4] Institute of Human Genetics, School of Medicine, Technical University of Munich, Munich, Germany
[5] Computational Health Center, Helmholtz Center Munich, Neuherberg, Germany

* Corresponding authors
gagneur@in.tum.de, mathias.wilhelm@tum.de

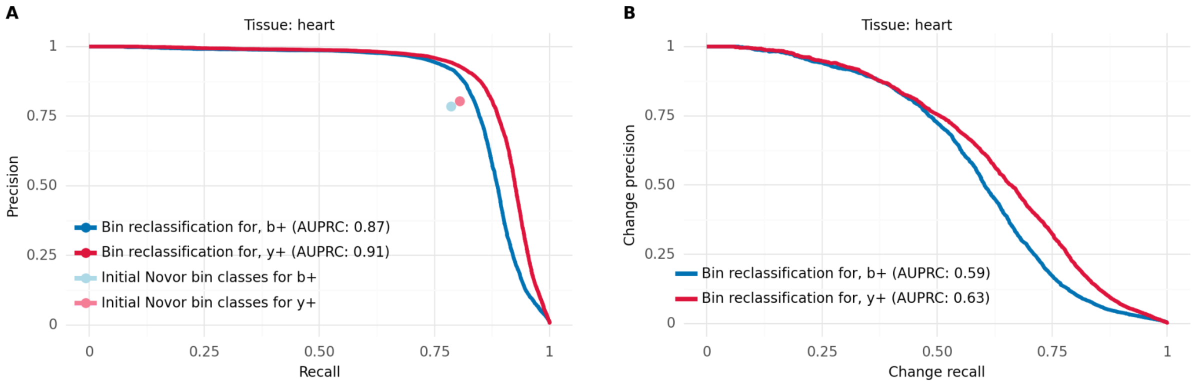

Supplementary Figure 1 | Bin reclassification performance for Novor-predicted peptides on the heart sample. A, Precision-recall curves for bin reclassification of b+ and y+ ion series after relabeling initial bin classes proposed by Novor on the test set of the heart sample compared to the precision and recall computed at bin level for the initial class labeling. **B,** Change-precision-recall curves at bin level for b+ and y+ ions on the test set of the heart sample after relabeling initial bin classes proposed by Novor on the heart sample.

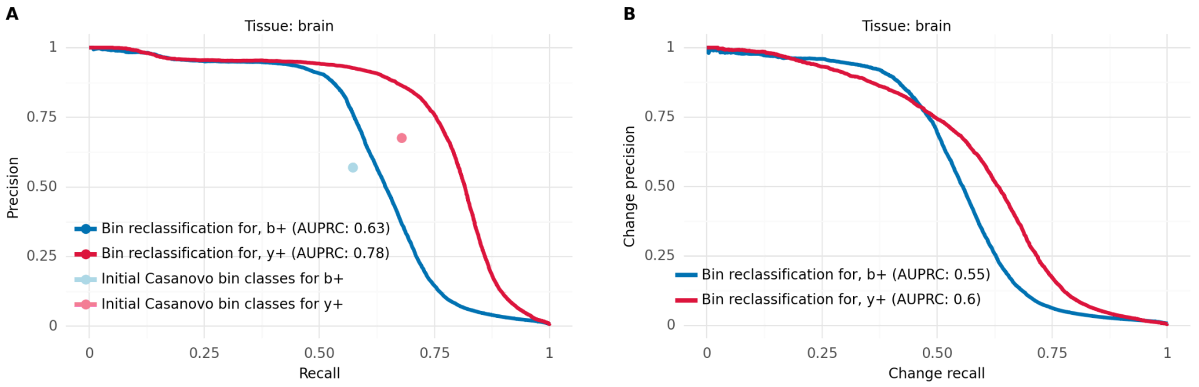

Supplementary Figure 2 | Bin reclassification performance for Casanovo-predicted peptides on the brain sample. A, Precision-recall curves for bin reclassification of b+ and y+ ion series after relabeling initial bin classes proposed by Casanovo on the test set of the brain sample compared to the precision and recall computed at bin level for the initial class labeling. **B,** Change-precision-recall curves at bin level for b+ and y+ ions on the test set of the heart sample after relabeling initial bin classes proposed by Casanovo on the brain sample.

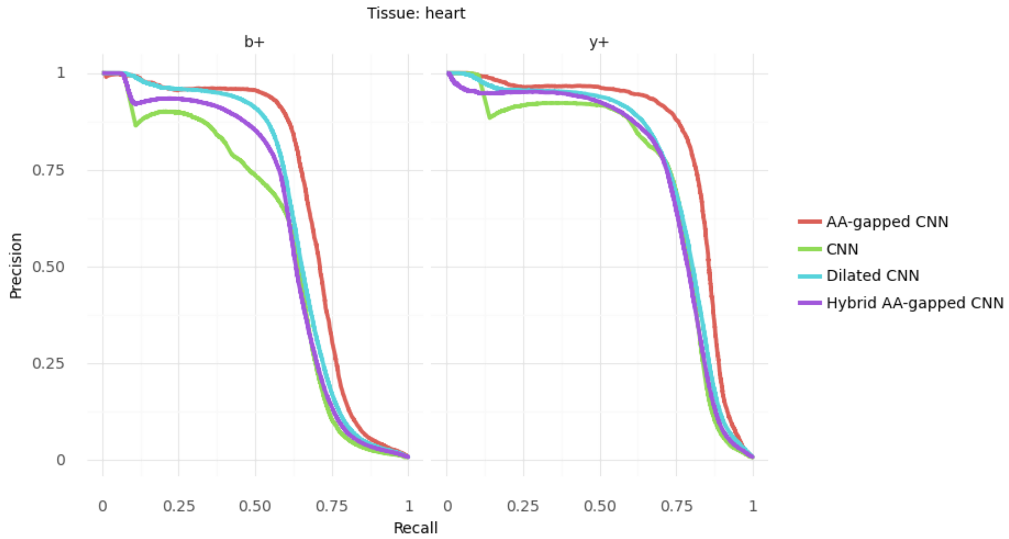

Supplementary Figure 3 | Comparison of different models for bin reclassification of Casanovo-predicted peptides on the heart sample. Precision-recall curves for bin reclassification of b+ and y+ ion series after relabeling initial bin classes proposed by Casanovo on the test set of the heart sample for different modeling approaches: a convolutional neural network (CNN) without any dilation, a CNN with regular dilations of size two, a hybrid model combining regular convolutional and AA-gapped layers, as well as a CNN with AA-gapped convolutions.

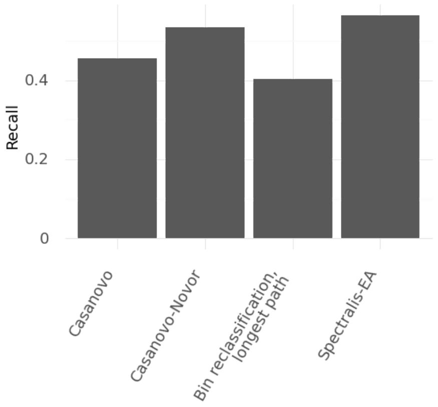

Supplementary Figure 4 | Performance comparison with different approaches. Recall at peptide level on the heart sample originally obtained from Casanovo, the combination of Casanovo and Novor peptides (Casanovo-Novor), the dynamic programming approach that determines the longest path in the graph with weights from the bin reclassification model and Spectralis-EA.

Supplementary Table 1 | Feature list and feature importances. Complete list of 114 features used as input for the random forest regressor to estimate the Levenshtein distance of a peptide candidate to the correct peptide sequence. The feature number indicates the order in which the features are passed to the model. The feature category indicates one of the three possible categories: similarity, counting, and bin reclassification-based features. The subcategory indicates which metric was used for feature computation or describes how the feature was computed. The specification indicates whether the feature was computed on all peaks or bins or whether only a subset was considered (only b- or y-ions or only bins with predicted change probability above a certain threshold) or whether a logarithmic transformation (log2) was applied prior to feature computation on the test set.

Category	Subcategory	Feature number	Specification	Importance
Similarity between matching experimental and PROSIT-predicted peaks	Spectral angle	1	all ions	0.0071
		2	b ions	0.0017
		3	y ions	0.0010
	Pearson correlation coefficient	4	all ions	0.0018
		5	b ions	0.0021
		6	y ions	0.0016
		7	all ions with log2 normalization	0.0158
		8	b ions with log2 normalization	0.0016
		9	y ions with log 2 normalization	0.0059
	Cosine similarity	10	all ions	0.0054
		11	b ions	0.0015
		12	y ions	0.0039
		13	all ions with log2 normalization	0.0045
		14	b ions with log2 normalization	0.0010
		15	y ions with log 2 normalization	0.0020
	Mean of absolute peak differences	16	all ions	0.0269
		25	b ions	0.0017
		34	y ions	0.0012
		43	all ions with log2 normalization	0.0018
		52	b ions with log2 normalization	0.0011
		61	y ions with log 2 normalization	0.0016
	Standard deviation of absolute peak differences	17	all ions	0.0030
		26	b ions	0.0018
		35	y ions	0.0017
		44	all ions with log2 normalization	0.0013

		53	b ions with log2 normalization	0.0010
		62	y ions with log 2 normalization	0.0024
	3rd quartile of absolute peak differences	18	all ions	0.0010
		27	b ions	0.0017
		36	y ions	0.0055
		45	all ions with log2 normalization	0.0014
		54	b ions with log2 normalization	0.0015
		63	y ions with log 2 normalization	0.0233
	2nd quartile of absolute peak differences	19	all ions	0.0008
		28	b ions	0.0017
		37	y ions	0.0015
		46	all ions with log2 normalization	0.0016
		55	b ions with log2 normalization	0.0011
		64	y ions with log 2 normalization	0.0105
	1st quartile of absolute peak differences	20	all ions	0.0007
		29	b ions	0.0015
		38	y ions	0.0020
		47	all ions with log2 normalization	0.0012
		56	b ions with log2 normalization	0.0010
		65	y ions with log 2 normalization	0.0027
	Minimum of absolute peak differences	21	all ions	0.0039
		30	b ions	0.0013
		39	y ions	0.0033
		48	all ions with log2 normalization	0.0016
		57	b ions with log2 normalization	0.0014
		66	y ions with log 2 normalization	0.0088
	Maximum of absolute peak differences	22	all ions	0.0012
		31	b ions	0.0013
		40	y ions	0.0011
		49	all ions with log2 normalization	0.0025
		58	b ions with log2 normalization	0.0016
		67	y ions with log 2 normalization	0.0171
		23	all ions	0.0018
		32	b ions	0.0018

	MSE of peak differences	41	y ions	0.0012
		50	all ions with log2 normalization	0.0012
		59	b ions with log2 normalization	0.0017
		68	y ions with log 2 normalization	0.0077
	Dot product ob peak differences	24	all ions	0.0081
		33	b ions	0.0015
		42	y ions	0.0013
		51	all ions with log2 normalization	0.0104
		60	b ions with log2 normalization	0.0020
		69	y ions with log 2 normalization	0.0045
	Spearman correlation coefficient	70	all ions	0.3163
		71	b ions	0.0010
		72	y ions	0.0013
		73	all ions with log2 normalization	0.3033
		74	b ions with log2 normalization	0.0010
		75	y ions with log 2 normalization	0.0013

Counting between matching experimental and PROSIT-predicted peaks	Number of peaks with non-zero experimental intensity and non-zero PROSIT-intensity	76	all ions	0.0036
		77	b ions	0.0033
		78	y ions	0.0036
	Number of peaks with non-zero experimental intensity and non-zero PROSIT-intensity relative to total amount of PROSIT-intensities	79	all ions	0.1397
		80	b ions	0.0087
		81	y ions	0.0081
	Number of peaks with non-zero experimental intensity and non-zero PROSIT-intensity relative to total amount of non-zero PROSIT-intensities	82	all ions	0.0101
		83	b ions	0.0021
		84	y ions	0.0003
	Number of peaks with non-zero experimental intensity and zero PROSIT-intensity	85	all ions	0.0039
		86	b ions	0.0006
		87	y ions	0.0051
	Number of peaks with non-zero experimental intensity and zero PROSIT-intensity relative to total amount of PROSIT-intensities	88	all ions	0.0054
		89	b ions	0.0019
		90	y ions	0.0061
	Number of peaks with non-zero	91	all ions	0.0052

	experimental intensity and zero PROSIT-intensity relative to total amount of non-zero PROSIT-intensities	92	b ions	0.0012
		93	y ions	0.0054
	Number of peaks with zero experimental intensity and non-zero PROSIT-intensity	94	all ions	0.0434
		95	b ions	0.0014
		96	y ions	0.0035
	Number of peaks with zero experimental intensity and non-zero PROSIT-intensity relative to total amount of PROSIT-intensities	97	all ions	0.0067
		98	b ions	0.0011
		99	y ions	0.0082
	Number of peaks with zero experimental intensity and non-zero PROSIT-intensity relative to total amount of non-zero PROSIT-intensities	100	all ions	0.0179
		101	b ions	0.0023
		102	y ions	0.0003
	Number of peaks with non-zero experimental intensity	103	all ions	0.0060
		104	b ions	0.0016
		105	y ions	0.0121
	Number of peaks with non-zero experimental intensity relative to total amount of PROSIT-intensities	106	all ions	0.0039
		107	b ions	0.0031
		108	y ions	0.0035
Binreclassification-based	Number of proposed bin changes above change probability threshold	109	threshold: 0.3	0.0335
		110	threshold: 0.4	0.0559
		111	threshold: 0.45	0.0828
		112	threshold: 0.5	0.1145
		113	threshold: 0.55	0.0795
		114	threshold: 0.6	0.1266

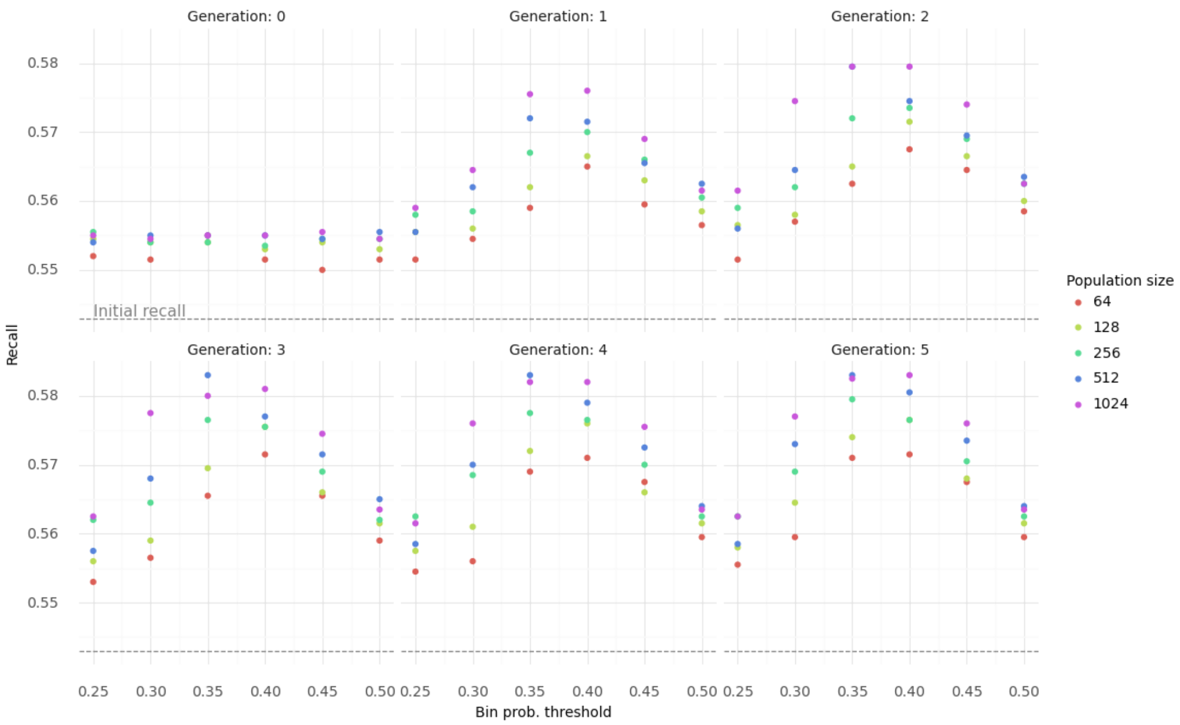

Supplementary Figure 5 | Performance comparison with different hyper-parameters. Peptide recall after running Spectralis-EA with different selections of bin probability threshold, number of generations and population size on a subset of the heart sample.

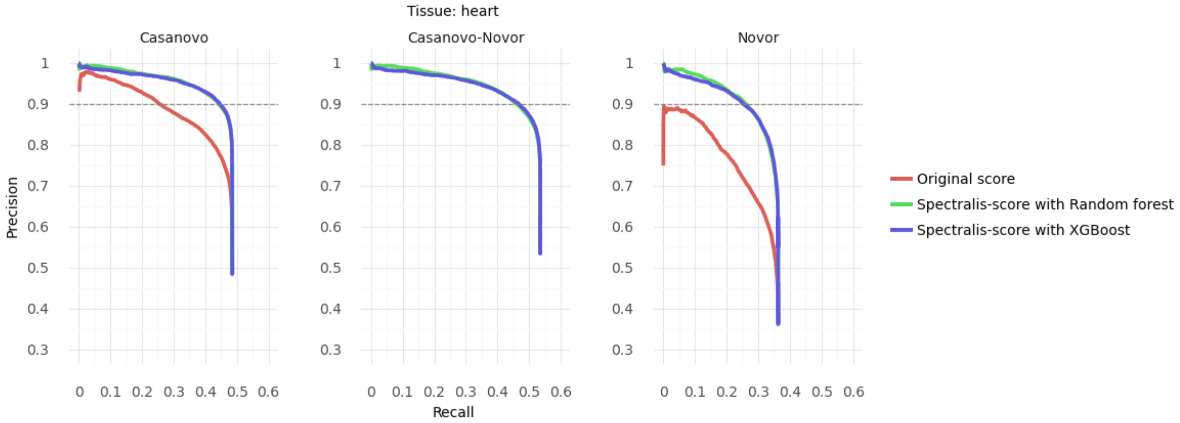

Supplementary Figure 6 | Levenshtein distance estimator performance on the heart sample with random forest and XGBoost model. Precision-recall curves at peptide level before and after rescoring peptide identifications by Novor and Casanovo on the heart sample with two trained regression models that estimate Levenshtein distances, including the precision and recall for the combination of Casanovo and Novor sequences (Casanovo-Novor).

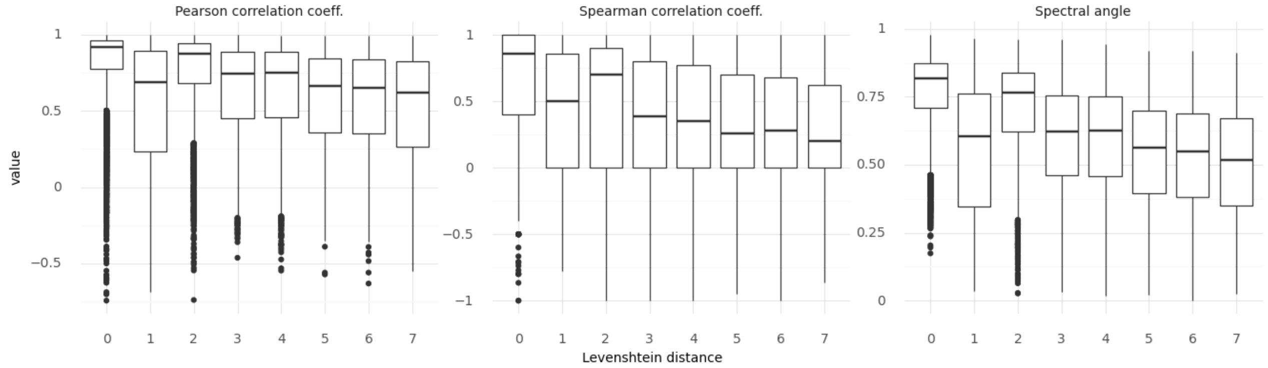

Supplementary Figure 7 | Distribution of features against different Levenshtein distances. Pearson and Spearman correlation coefficients, as well as spectral angles against Levenshtein distance of peptide identifications by Novor and Casanovo to the correct peptide sequence by MaxQuant across all 30 human samples for initial Levenshtein distances smaller than 8.

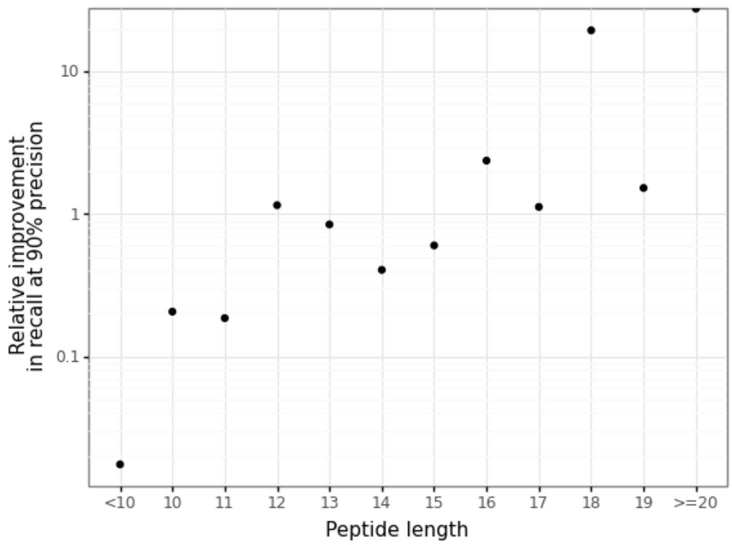

Supplementary Figure 8 | Relative performance improvement on the representative sample heart by peptide length. Relative improvement in recall at 90% precision stratified by peptide length for peptides proposed by Casanovo on the heart sample.

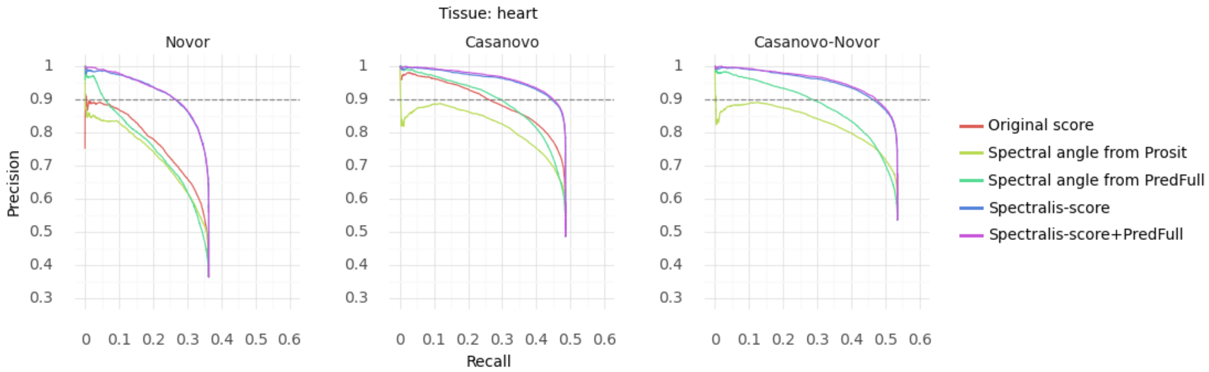

Supplementary Figure 9 | Levenshtein distance estimator performance on the representative sample heart benchmarked against Spectral Angles by Prosit and PredFull. Precision-recall curves for peptide identification by Novor (left), Casanovo (middle) and the combination of Casanovo and Novor sequences (Casanovo-Novor, right) on the heart sample sorted by spectral angle using spectrum predictions from Prosit (green) or PredFull (turquoise), for Spectralis-score (dark blue), and for a score integrating Spectralis-score and the spectral angle using spectrum predictions from PredFull (magenta). Moreover, peptides sorted by the original score of Novor (left) and Casanovo (middle) are shown in red.

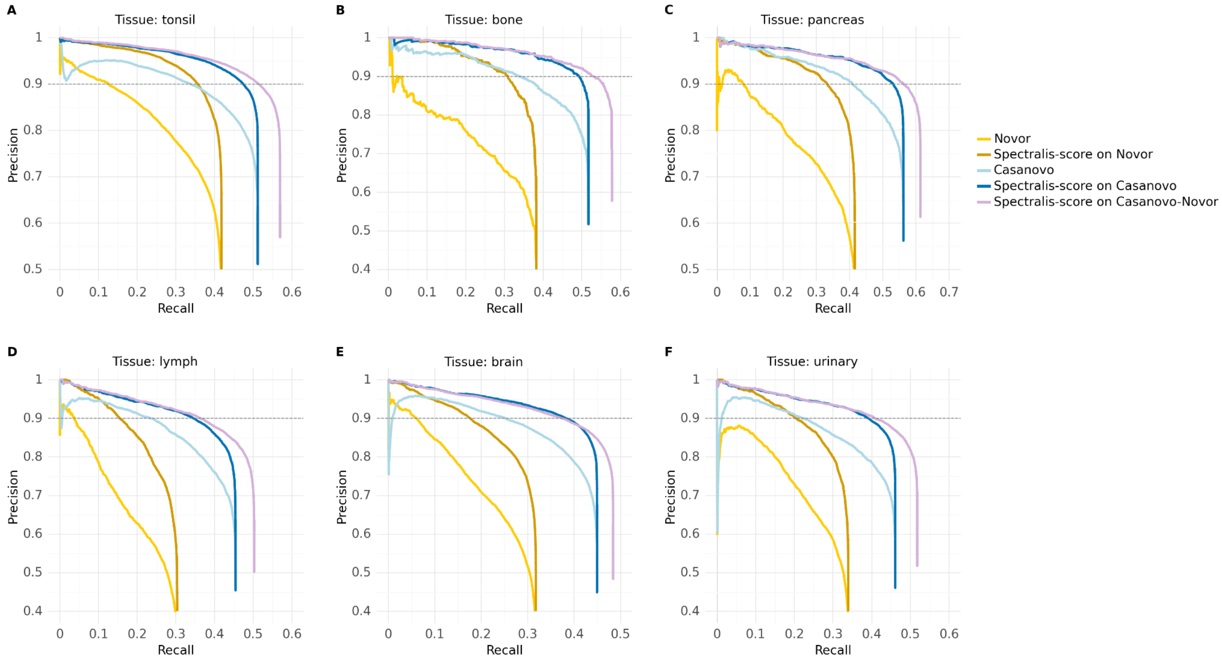

Supplementary Figure 10 | Levenshtein distance estimator performance on the three best-performing and three worst-performing samples. Precision-recall curves at peptide level before and after rescoring peptide identifications by Novor and Casanovo on the three best-performing samples (A-C) and the three worst-performing samples (D-F) with the trained regression model that estimates Levenshtein distances, including the precision and recall for the combination of Casanovo and Novor sequences (Casanovo-Novor).

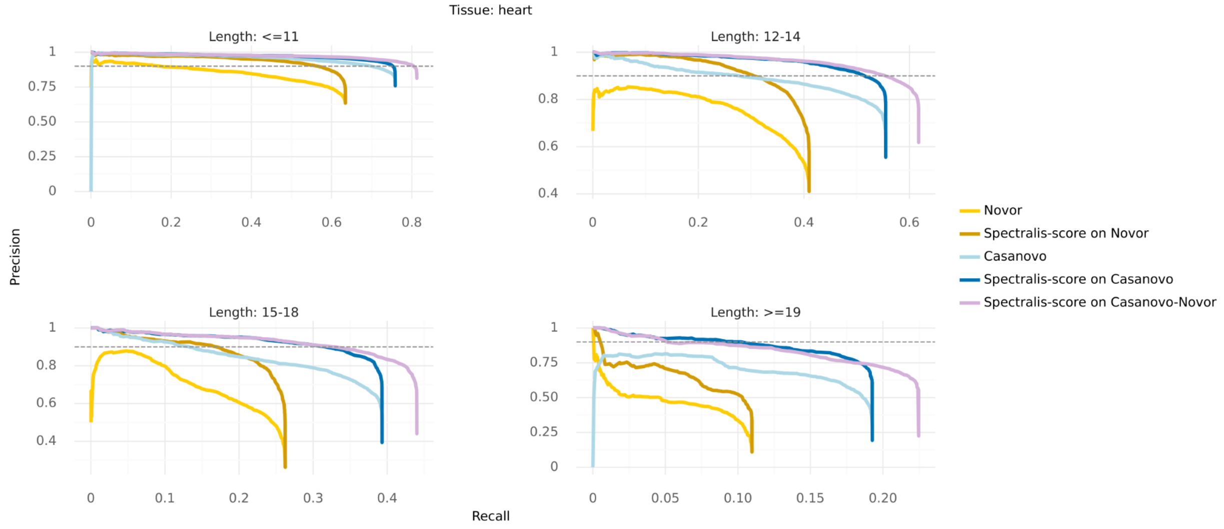

Supplementary Figure 11 | Levenshtein distance estimator performance on the heart sample stratified by peptide length. Precision-recall curves at peptide level before and after rescoring peptide identifications by Novor and Casanovo on the heart sample using the Levenshtein distance estimator, including the precision and recall for the combination of Casanovo and Novor sequences (Casanovo-Novor), stratified by peptide length ranges.

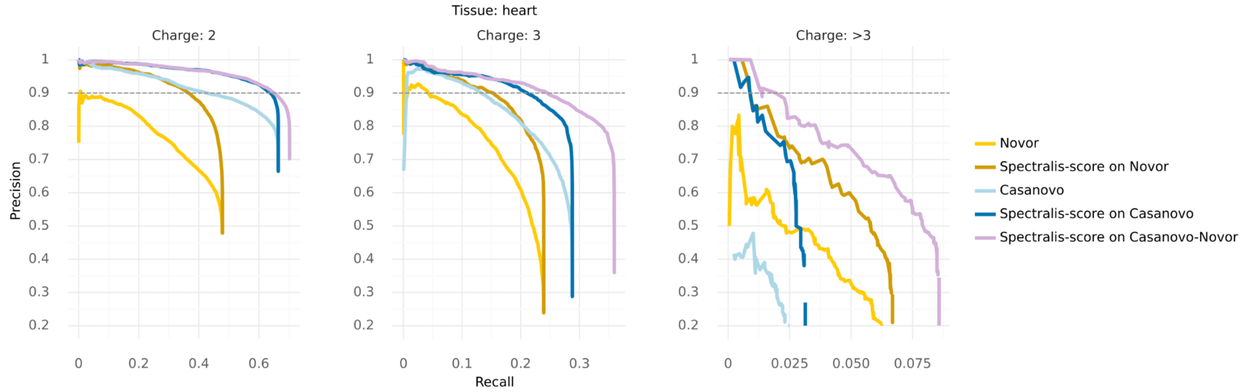

Supplementary Figure 12 | Levenshtein distance estimator performance on the heart sample stratified by precursor charge state. Precision-recall curves at peptide level before and after rescoring peptide identifications by Novor and Casanovo on the heart sample using the Levenshtein distance estimator, including the precision and recall for the combination of Casanovo and Novor sequences (Casanovo-Novor), stratified by peptide precursor charge states.

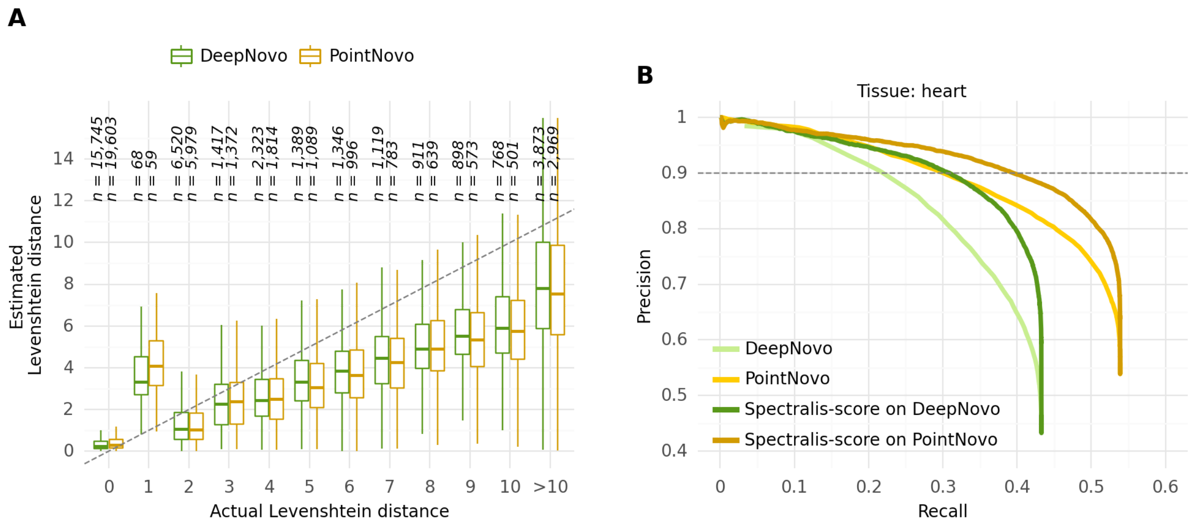

Supplementary Figure 13 | Levenshtein distance estimator performance on the heart sample for peptides predicted by DeepNovo and PointNovo. A, Estimated against actual Levenshtein distances of incorrect peptide identifications by DeepNovo and PointNovo to the correct peptide sequence by MaxQuant for the heart sample. **B,** Precision-recall curves at peptide level before and after rescoring peptide identifications by DeepNovo and PointNovo on the heart sample with the regression model trained to estimate Levenshtein distances.

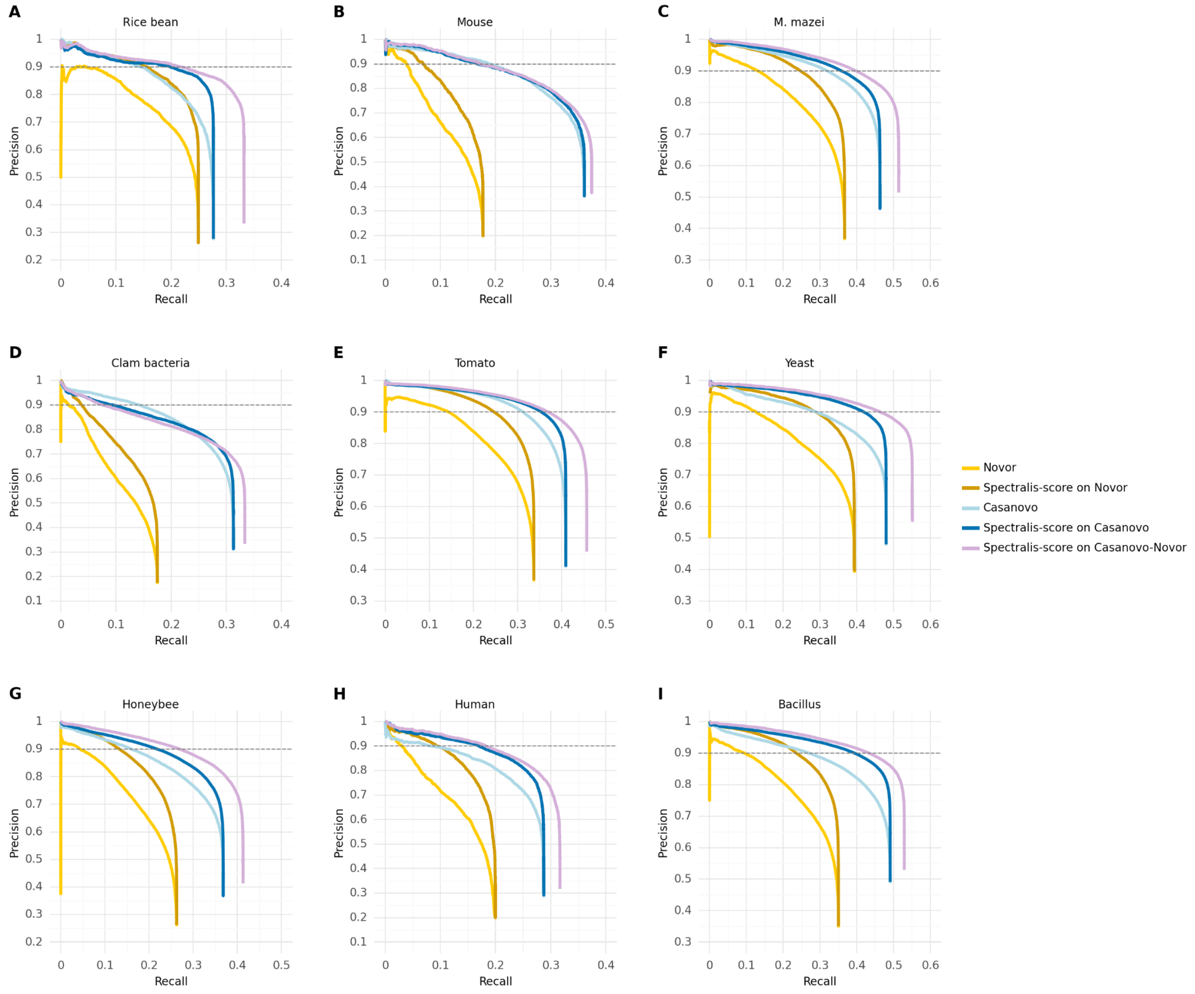

Supplementary Figure 14 | Levenshtein distance estimator performance on the nine-species dataset. Precision-recall curves at peptide level before and after rescoring peptide identifications by Novor and Casanovo on the nine-species dataset originally introduced by Tran et al. using the Levenshtein distance estimator, including the precision and recall for the combination of Casanovo and Novor sequences (Casanovo-Novor).

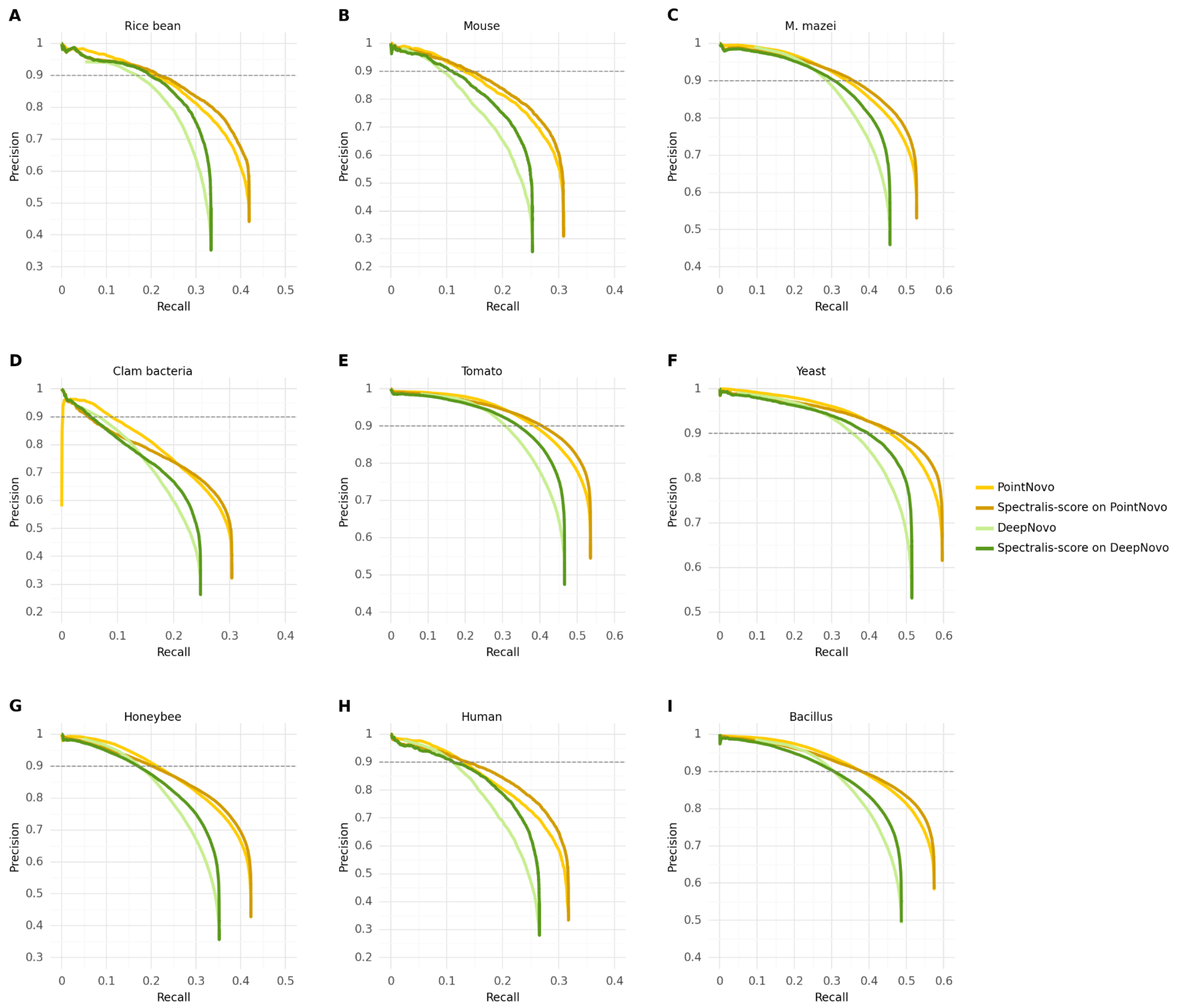

Supplementary Figure 15 | Levenshtein distance estimator performance on the nine-species dataset. Precision-recall curves at peptide level before and after rescoring peptide identifications by DeepNovo and PointNovo on the nine-species dataset originally introduced by Tran et al. using the Levenshtein distance estimator.

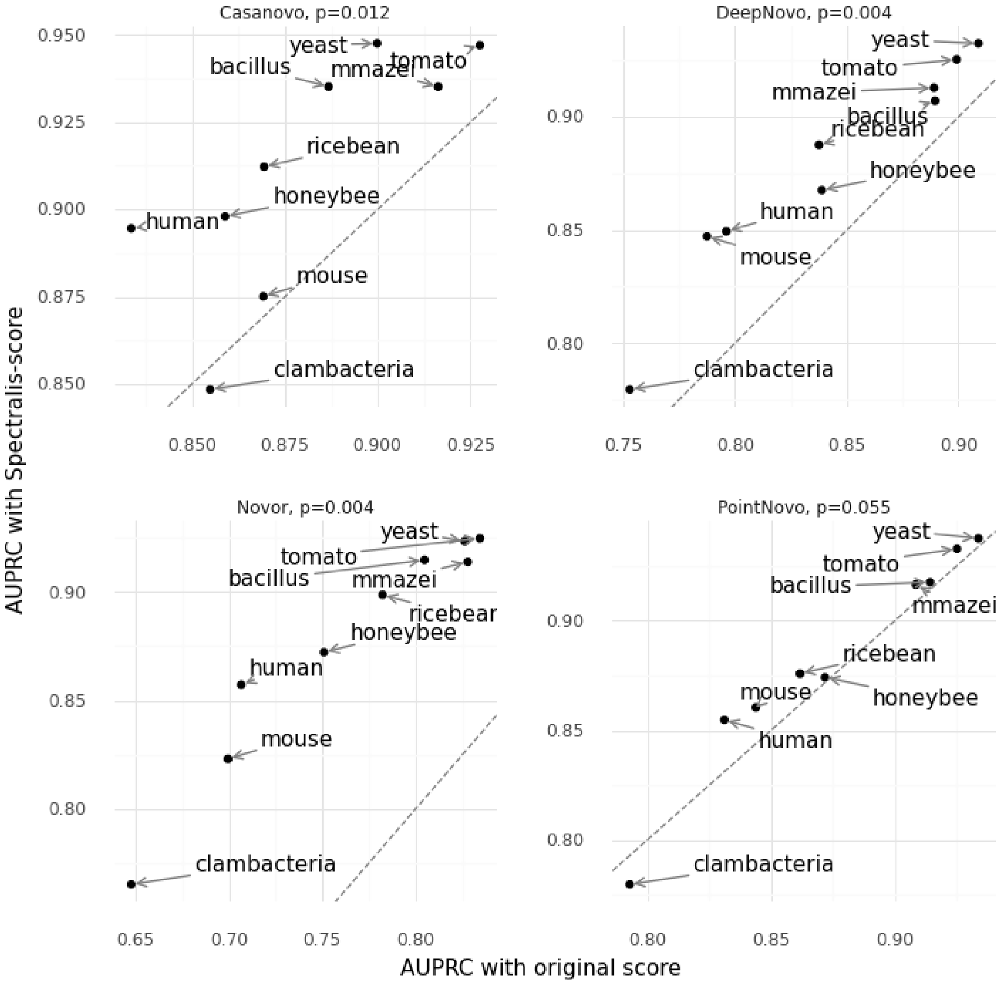

Supplementary Figure 16 | Performance comparison on the nine-species dataset before and after rescoring. Average precision-recall (AUPRC) at peptide level before and after rescoring peptide identifications by Casanovo, Novor, DeepNovo and PointNovo on the nine-species dataset originally introduced by Tran et al. using the Levenshtein distance estimator. Statistical significance from paired Wilcoxon test (n = 9).

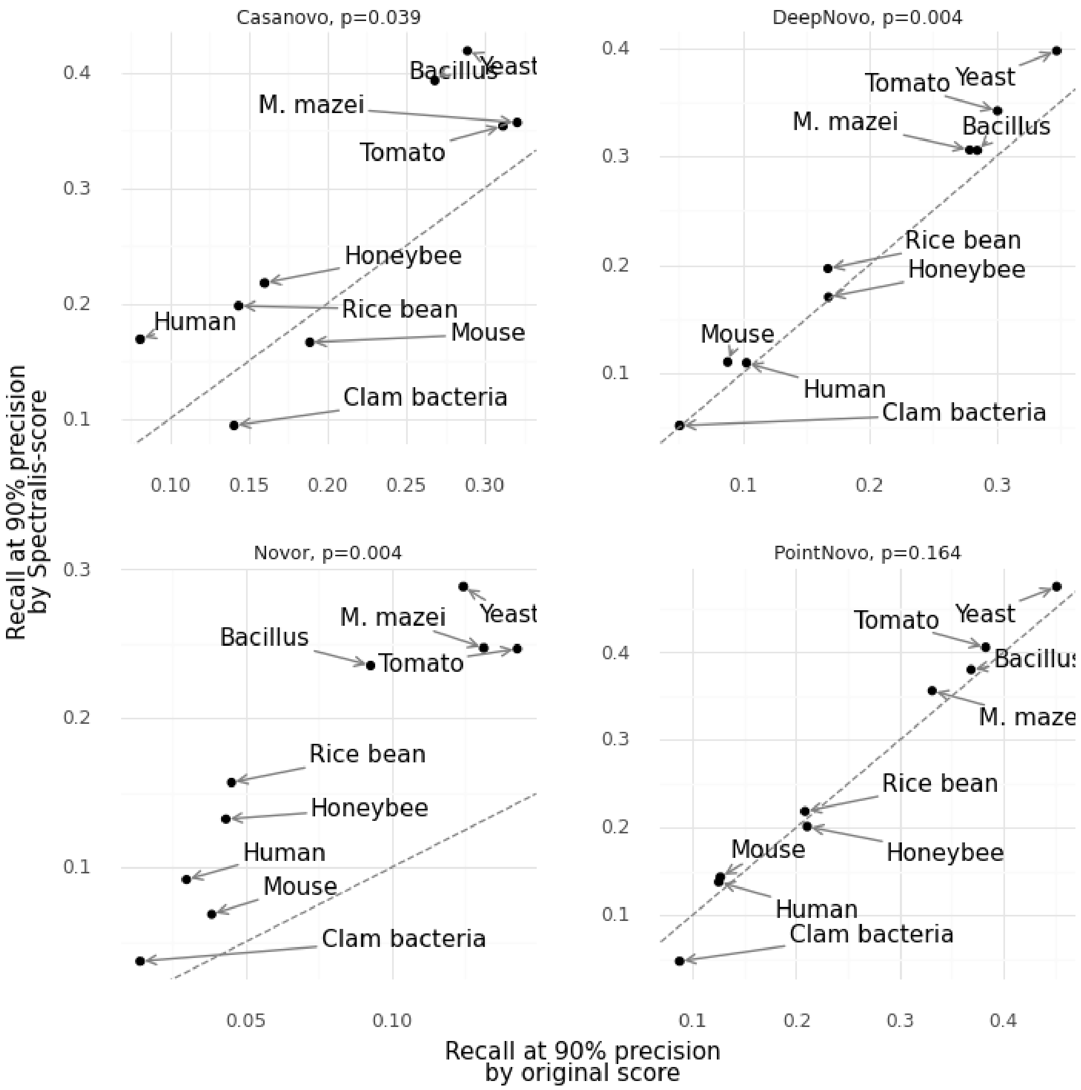

Supplementary Figure 17 | Recall comparison on the nine-species dataset before and after rescoring. Recall at 90% precision at peptide level before and after rescoring peptide identifications by Casanovo, Novor, DeepNovo and PointNovo on the nine-species dataset originally introduced by Tran et al. using the Levenshtein distance estimator. Statistical significance from paired Wilcoxon test (n = 9).

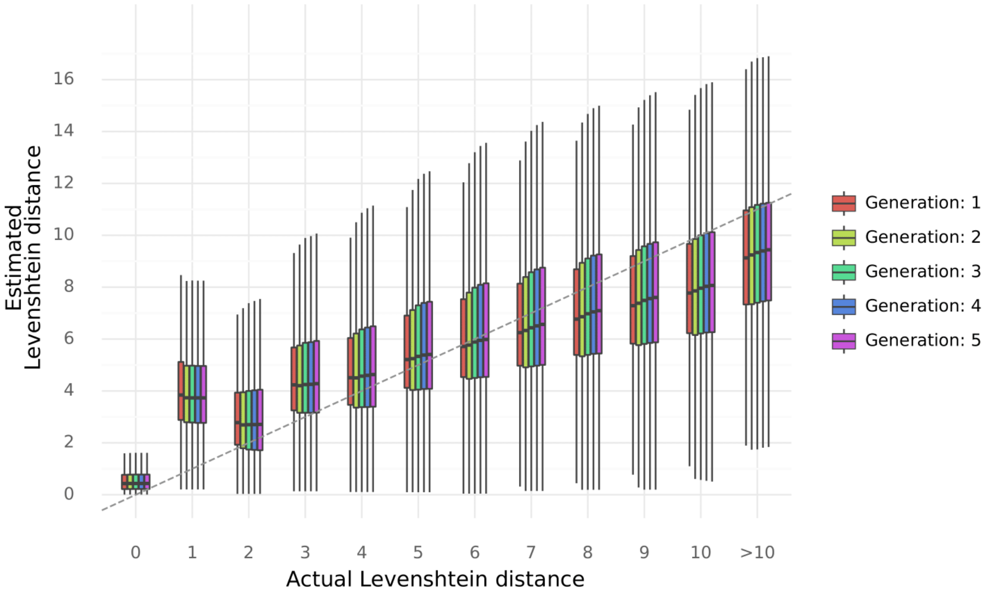

Supplementary Figure 18 | Levenshtein distance estimator performance on different generations of Spectralis-EA. Estimated against actual Levenshtein distances of peptide candidates proposed by Spectralis-EA at different generations.

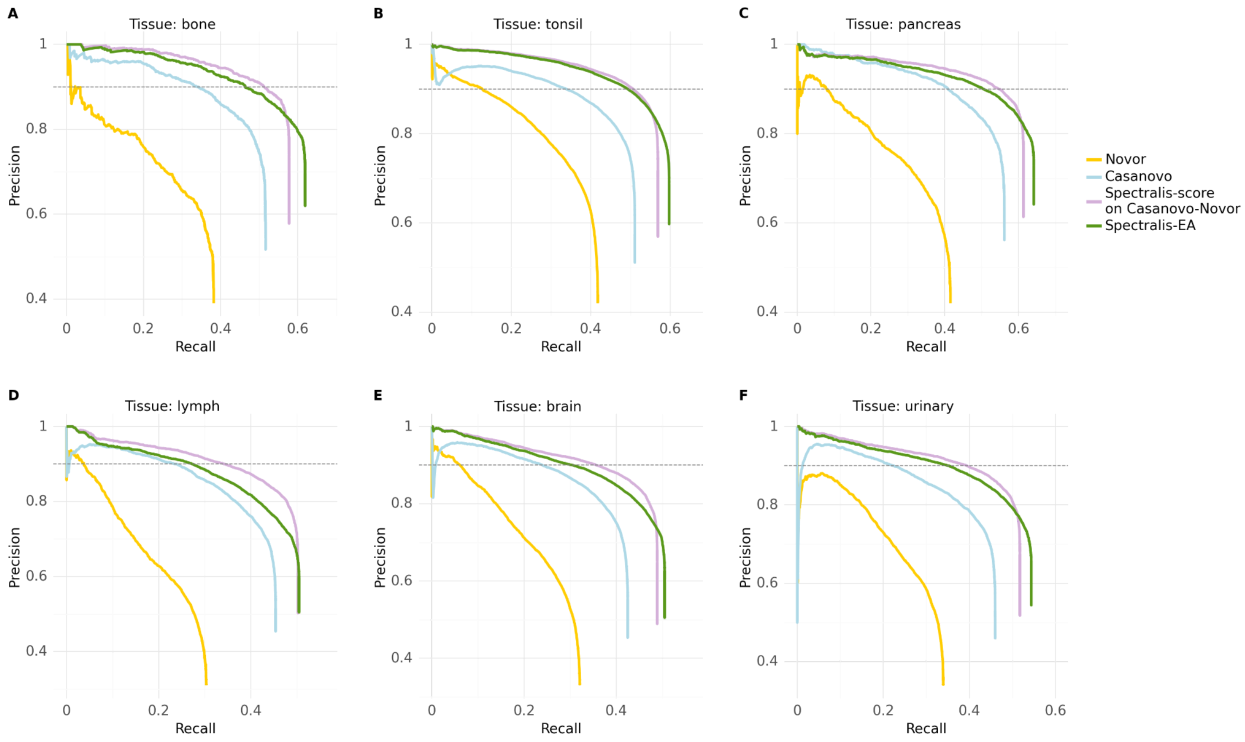

Supplementary Figure 19 | Spectralis-EA performance on the three best-performing and three-worst performing samples. Precision-recall curves of identifications at peptide level for Novor, Casanovo, and Spectralis-EA on the three best-performing samples (A-C) and the three worst-performing samples (D-F) including the precision and recall for the combination of Casanovo and Novor sequences (Casanovo-Novor).

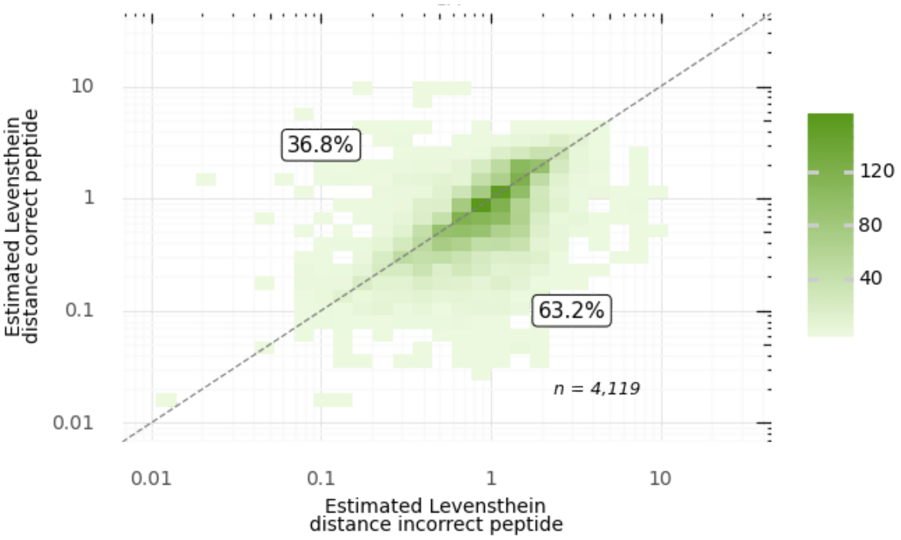

Supplementary Figure 20 | Comparison of estimated Levenshtein distances for correct and incorrect peptides with a Levenshtein distance of two. Estimated Levenshtein distances of incorrect peptides against the estimated Levenshtein distances of the corresponding correct peptide sequences for a given spectrum across all 30 samples for peptide sequences with an actual Levenshtein distance of two to the correct peptide.

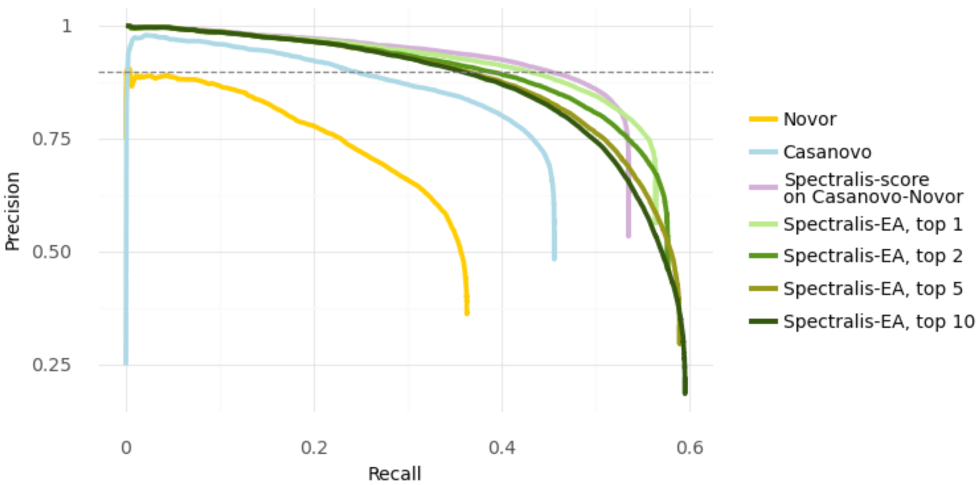

Supplementary Figure 21 | Spectralis performance on the heart sample including multiple peptide predictions per spectrum. Precision-recall curves of identifications at peptide level for Novor, Casanovo and Spectralis-EA on the test set of the heart sample, including the precision and recall for the combination of Casanovo and Novor sequences (Casanovo-Novor) and the Spectralis-EA returning the top 1,2,5 and 10 best-scored peptide candidates per spectrum.

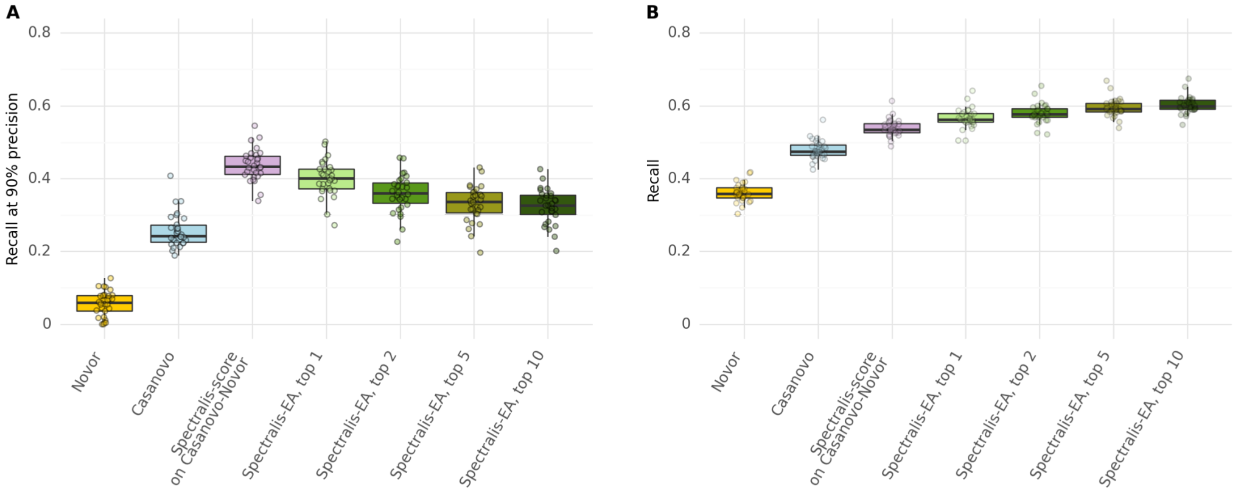

Supplementary Figure 22 | Spectralis performance on all samples including multiple peptide predictions per spectrum. A, Recall at 90% precision for Novor, Casanovo, Spectralis-score on the combination of Casanovo and Novor (Casanovo-Novor) and Spectralis-EA returning the top 1,2,5 and 10 best score peptide candidates on the test sets of all 30 samples. **B,** Overall recall for Novor, Casanovo, Spectralis-score Casanovo-Novor, and Spectralis-EA returning the top 1,2,5 and 10 best score peptide candidates on the test sets of all 30 samples.

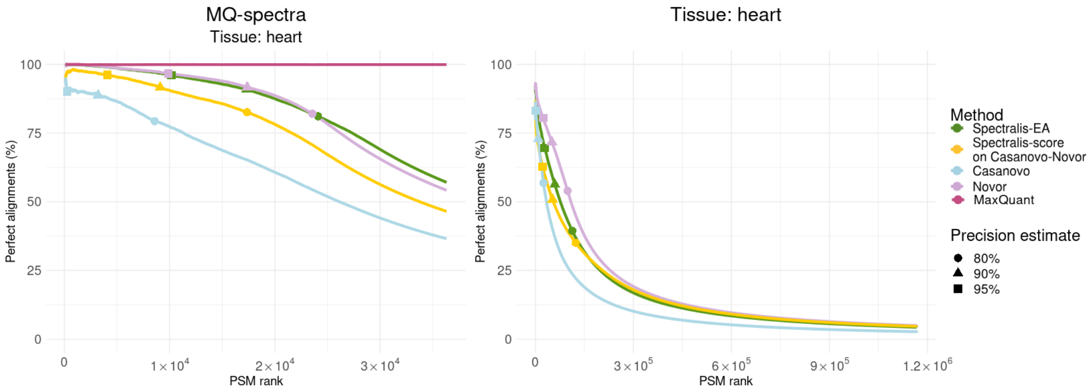

Supplementary Figure 23 | Percentage of perfect alignments for heart sample. Left: percentage of perfect alignments queried on peptide sequences by MaxQuant, Novor, Casanovo and Spectralis-score on Casanovo-Novor and Spectralis-EA against a set of known and predicted gene translations using blastp on the set of spectra identified by MaxQuant (MQ-spectra, n = 36,312) of the heart sample. For clarity, the first 100 peptides are omitted. Right: same as left, but for spectra not identified by MaxQuant (non-MQ-spectra, n = 1,167,029).

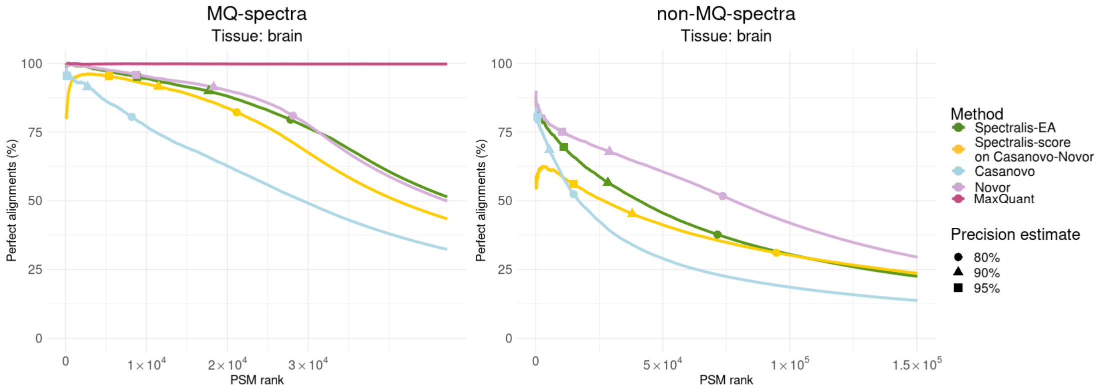

Supplementary Figure 24 | Percentage of perfect blastp matches for brain sample. Left: Percentage of perfect alignments queried on peptide sequences by MaxQuant, Novor, Casanovo and Spectralis-score on Casanovo-Novor and Spectralis-EA against a set of known and predicted gene translations using blastp on the set of spectra identified by MaxQuant (MQ-spectra, n = 47,181) of the brain sample. For clarity, the first 100 peptides are omitted. Right: same as left, but for spectra not identified by MaxQuant (non-MQ-spectra) showing only the top 150,000 ranked peptide candidates of each method (out of 1,040,253).

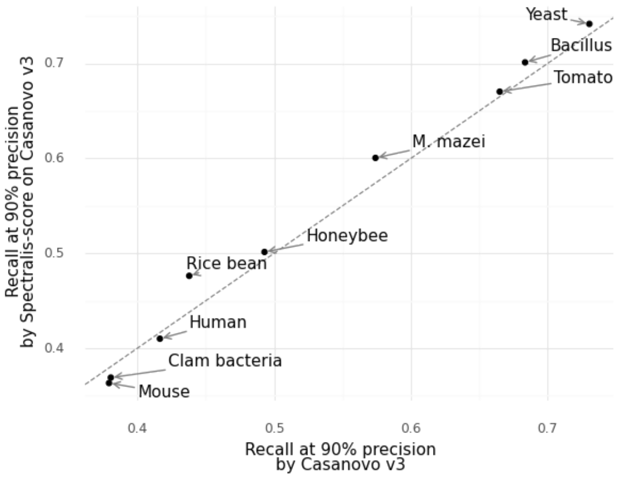

Supplementary Figure 25 | Levenshtein distance estimator performance on Peptides by Casanovo v.3.2.0 on the nine species dataset. Recall at 90% precision before and after rescoring peptide identification by the latest version of Casanovo (v3.2.0) on the nine species dataset originally introduced by Tran et al. with the trained regression model that estimates Levenshtein distances.
